# Completion of secondary education among children born in deprived neighbourhoods in Amsterdam and Stockholm: The role of residential mobility and exposure to neighbourhood deprivation

**DOI:** 10.1177/00420980261442414

**Published:** 2026-05-20

**Authors:** Joeke Kuyvenhoven

**Affiliations:** 1 Netherlands Interdisciplinary Demographic Institute (NIDI)-KNAW/University of Groningen, The Netherlands; 2 Department of Human Geography, Stockholm University, Sweden; 3 Department of Human Geography, Planning and International Development, University of Amsterdam, The Netherlands

**Keywords:** childhood, educational attainment, moving frequency, neighbourhood deprivation, residential mobility, 儿童期, 教育成就, 迁居频率, 社区贫困, 居住迁移

## Abstract

Residential mobility during childhood is an important life event altering a child’s environment with possible long-term consequences. While moving frequently may be particularly disruptive, moving to a better environment might outweigh the disruptive effects. This study simultaneously examines *frequency of moving* and *exposure to neighbourhood deprivation*, and the association with completion of secondary education for children born in socio-economically deprived neighbourhoods in Amsterdam and Stockholm. Using full-population register data from the Netherlands and Sweden, residential mobility is studied from birth until age 16. Completion of secondary education is analysed at age 21, comparing children who do not move to children with diverse mobility patterns using propensity-weighted logistic regression models. Results indicate that the positive association between moving to a better neighbourhood and completion of secondary education is primarily explained by the better socio-economic position of upwardly mobile families. Frequent moving with long-term exposure to neighbourhood deprivation decreases the likelihood of completing secondary education, particularly in Stockholm. Children in intact families benefit more from upward mobility, while frequent moving is more detrimental for children in non-intact families. The study concludes that combinations of positive and negative childhood circumstances influence educational disparities, in which residential mobility, exposure to neighbourhood deprivation, and family stability play a crucial role in accumulations of (dis)advantage over a child’s life course.

## 摘要

儿童期居住迁移是一项重要的生活事件，它会改变儿童的成长环境，且可能产生长期后果。尽管频繁迁居可能具有尤为突出的干扰性，但迁至更优质环境带来的影响，或许能盖过这种干扰效应。本研究同时考察了在阿姆斯特丹和斯德哥尔摩社会经济贫困社区出生儿童的迁居频率、社区贫困暴露度，以及二者与中等教育完成情况之间的关联。本研究借助荷兰与瑞典的全人口登记数据，考察了研究对象从出生至 16 岁期间的居住迁移情况。本研究在研究对象 21 岁时分析其中等教育完成情况，采用倾向得分加权逻辑回归模型，将未迁居儿童与具有不同迁居模式的儿童进行对比。研究结果表明，迁至更优质社区与中等教育完成之间的正向关联，主要可由实现向上流动的家庭所具备的更优社会经济地位来解释。频繁迁居且长期暴露于社区贫困环境中，会降低儿童完成中等教育的可能性，这一效应在斯德哥尔摩尤为显著。完整家庭的儿童能从向上流动中获益更多，而频繁迁居对非完整家庭的儿童具有更强的不利影响。本研究得出结论，儿童期积极与消极境遇的组合会影响教育差距，其中居住迁移、社区贫困暴露度与家庭稳定性，在儿童整个生命历程的（优）劣势累积过程中发挥着关键作用。

## Introduction

It is widely recognised that a child’s social environment of family, peers, school and neighbourhood plays an important role in shaping educational opportunities. Moving during childhood alters a child’s environment, which may have long-term consequences for educational attainment (Coley and Kull, 2016). On the one hand, moving is stressful and disruptive, and may hamper educational progress especially when experienced repeatedly (Mollborn et al., 2018). There is increasing evidence that moving frequently is negatively associated with educational outcomes (Haugan and Myhr, 2019; Hutchings et al., 2013; Tønnessen et al., 2016). On the other hand, moving may be a planned decision of parents to improve living conditions (Coulton et al., 2012) and moving out of deprived neighbourhoods might be beneficial for children’s educational attainment in the long run (Chetty et al., 2016; Hango, 2006; Mollborn et al., 2018).

The non-random selection into patterns of mobility adds to the complexity of understanding its consequences, as factors associated with the move and neighbourhood context also impact children’s educational outcomes (Garboden et al., 2017). Mobility is higher among children in deprived neighbourhoods (Kuyvenhoven et al., 2025; Vogel and Zwiers, 2018), but varies by family structure and background (Coulton et al., 2012). Moves to improve living conditions are usually made by better-off households, while frequent moves out of necessity due to family instability or financial or housing insecurities are often made by lower-income households (Coulton et al., 2012).

This study contributes to the literature by integrating insights from residential mobility and neighbourhood effects research within a life-course framework acknowledging the interrelatedness of childhood moves and neighbourhood context affecting accumulations of (dis)advantage. Building on this framework, the study addresses three gaps. First, the consequences of moving frequency and neighbourhood deprivation throughout childhood for completion of secondary education are studied simultaneously. Second, it aims to gain insights into those consequences among a comparable group of children by focussing on those born in socio-economically deprived neighbourhoods while minimising selection bias using propensity-score methods. Third, heterogeneity in effects is assessed for children in intact and non-intact families acknowledging the cumulative impact of disruptions across life domains.

The study focuses on the metropolitan areas of Amsterdam and Stockholm, using longitudinal full-population register data for both cities. While a comprehensive city comparison is beyond the scope of this study, studying and comparing both capitals can provide insights into consequences of childhood residential mobility and exposure to neighbourhood deprivation within distinct urban contexts. Furthermore, it contributes to a more general understanding of these processes among a highly mobile population.

## A dynamic longitudinal perspective on children and their environment

Across disciplines, children’s socialisation and development is increasingly viewed as resulting from a complex and dynamic interplay between a child and the multiple interrelated environments in which they are embedded (Elder and Shanahan, 2007). Those environments, including the family, peers, neighbourhood and school, often change when a child moves house. Moving is thus not only a stressful event in itself, but also changes the context in which a child develops and grows up (Anderson et al., 2014). Integrating insights from the residential mobility and neighbourhood effects literature within a life-course framework underlines how interrelated processes affect individual life chances through accumulations of (dis)advantages over time and life domains. Within this framework, five interrelated dimensions are identified and discussed: selective mobility patterns, neighbourhood mobility, moving frequency, heterogeneity in mobility effects, and timing and distance of the move.

### Selective mobility patterns

Residential (im)mobility and neighbourhood sorting are not random processes but related to sociodemographic characteristics, life course trajectories and events that might trigger a move, individual preferences and resources, and structural opportunities and constraints (Mulder and Hooimeijer, 1999). Children living in deprived neighbourhoods are found to have higher mobility rates (Kuyvenhoven et al., 2025; Vogel and Zwiers, 2018), but mobility also varies by family structure and background (Coulton et al., 2012). Upward neighbourhood mobility is more common among higher-income and higher-educated families, as moving out of deprived areas requires financial and social resources (Clark et al., 2014; Goldsmith et al., 2017). On the other hand, mobility might reflect family, financial or housing instability (Coulton et al., 2012). Negative events such as union dissolution or job loss are found to be related to moves within or towards deprived neighbourhoods, and frequent residential moves (Clark, 2016; Gambaro et al., 2017; Morris, 2017; South et al., 1998). Children moving frequently are also more likely to live in lower socio-economic status families and single-parent households (Long, 1992; Murphey et al., 2012). While immobility may reflect stability and choice, it may also result from constraints and a lack of resources to make a desired move (Coulton et al., 2012; Kemppainen et al., 2020). Theories of spatial assimilation, structural constraints, and preferences to live among residents with a similar background suggest a pattern of high-income and non-migrant residents leaving deprived areas, while low-income and migrant residents stay (Sampson and Sharkey, 2008; Sharkey, 2013).

Selective patterns of (im)mobility are a major challenge in both studies on residential mobility effects (Garboden et al., 2017) and neighbourhood effects (Hedman and Van Ham, 2012), as factors associated with mobility and the residential neighbourhood also impact educational outcomes. With the availability of longitudinal data and methodological advancements, scholars increasingly attempt to correct for selection by modelling strategies such as propensity score methods, instrumental variables and fixed effects (Garboden et al., 2017; Hedman and Van Ham, 2012). Such studies indeed demonstrate the complexity of residential mobility and neighbourhood effects, generally pointing to results being biased by selection (Garboden et al., 2017).

### Upward neighbourhood mobility

Traditionally, residential mobility was viewed as a positive experience triggered by employment or changing housing needs related to the life-cycle in which moves were often assumed to be planned and made within a structure of opportunities, choice and resources (Lennon et al., 2016). From this perspective, the improvements in housing and neighbourhoods due to a move might benefit children’s education (Hango, 2006; Mollborn et al., 2018). Although neighbourhood effects literature remains inconclusive about the relative importance of the neighbourhood for education (see Nieuwenhuis and Hooimeijer, 2016 for an overview), there is increasing evidence that long-term exposure to neighbourhood deprivation has a detrimental impact on educational outcomes (Nieuwenhuis et al., 2021; Sampson et al., 2008; Wodtke et al., 2011). The time lived in an affluent neighbourhood might compensate for the time lived in a deprived neighbourhood, and moving out of a deprived neighbourhood may thus positively impact educational attainment (Chetty et al., 2016). However, when accounting for observed and unobserved family characteristics, Tarkiainen et al. (2024) find for the Finnish context no effect of exposure to neighbourhood deprivation on educational achievements, except for a strong but non-significant negative association among children with lower educated parents.

Empirical evidence on upward neighbourhood mobility and educational outcomes is limited and mainly builds on evaluations of the large-scale ‘Moving to Opportunity’ experiment in the US, in which families in deprived neighbourhoods were randomly assigned to move to lower poverty neighbourhoods. Those studies show mixed findings. Moving to a less deprived neighbourhood did not improve medium-term educational achievements, even for children moving before the age of six (Gennetian et al., 2012). However, moving out of deprivation did positively affect long-term educational attainment, with increased college attendance when the move occurred before the age of 13 (Chetty et al., 2016). Furthermore, females were found to benefit more from a move out of a deprived neighbourhood in the medium-term (Gennetian et al., 2012), while no gender differences were observed in long-term educational outcomes (Chetty et al., 2016). Non-experimental studies find that moving negatively impacts educational attainment regardless of the destination neighbourhood (Cordes et al., 2016; Metzger et al., 2015; Sweet et al., 2018).

A potential reason for the inconsistency in findings relates to the duration of exposure and accumulations of (dis)advantage. Most studies neither take into account the number of moving experiences in childhood nor the duration of exposure to a neighbourhood. The extent to which upward neighbourhood mobility has a positive impact may depend on the duration of exposure to both deprived and more affluent neighbourhoods and thus relate to the age at moving. Furthermore, while a move to an improved environment may outweigh the disruptive mobility effect, this may not apply when residential mobility is experienced repeatedly or coincides with disruptions across other life domains (Mollborn et al., 2018).

**Hypothesis 1**: Low residential mobility (one or two moves) with shorter duration of exposure to neighbourhood deprivation increases the likelihood of completing secondary education compared to staying in a deprived neighbourhood throughout childhood.

### Frequent residential mobility

There is increasing attention for moves that are unintended and related to negative circumstances or events, such as financial or housing instability, job loss or union dissolution (Clark, 2016). Moreover, moving house is often a source of stress and disruption for a child and it has been theorised that particularly frequent moving negatively impacts children. First, grounded in family stress theory, the instability of repeated residential moves with little time to adjust to a changing environment is a particular risk factor for children (Mollborn et al., 2018). Secondly, frequent moving is often related to other family problems, instabilities and negative life-events and more often experienced by families in socio-economically disadvantaged situations (Long, 1992). Frequent moving might thus be an added stressor for children in already instable and vulnerable situations.

Empirical studies indeed show that frequent moving is associated with worse educational outcomes (Haugan and Myhr, 2019; Hutchings et al., 2013; Tønnessen et al., 2016). However, some studies suggest that such negative associations can be explained by selective mobility or an accumulation of family instability (Tucker et al., 1998; Vidal and Baxter, 2018). Most studies focus primarily on moving frequency without taking into consideration the types of neighbourhoods children live in throughout childhood. Frequent moving might be particularly detrimental in combination with long-term exposure to deprivation, adding to an accumulation of disadvantage.

**Hypothesis 2**: High residential mobility (three or more moves) decreases the likelihood of completing secondary education compared to staying in a deprived neighbourhood throughout childhood. This negative association is expected to be more pronounced in combination with long-term exposure to neighbourhood deprivation.

### Heterogeneity in mobility-effects by family stability

Research on residential mobility has paid much attention to the role of parental union dissolution as both a factor explaining mobility and adding to the negative consequences of mobility. Union dissolution is consistently found to result in increased levels of residential mobility, especially to more deprived neighbourhoods (Clark et al., 2014; South et al., 1998). Children in non-intact families move more often due to changes in the family structure and the resulting decreased financial resources (South et al., 1998). Such disruptions across multiple domains puts children in non-intact families at particular risk (Mollborn et al., 2018). Research on heterogeneity in mobility effects indeed suggests a stronger negative impact of moving for educational outcomes among children in non-intact families (Haugan and Myhr, 2019; Scanlon and Devine, 2001; Tucker et al., 1998). Children in non-intact families may benefit less from upward mobility as it might not compensate for multiple disruptions across life domains, while the detrimental impact of frequent moves may be more severe for this group as this adds to accumulated disadvantage.

**Hypothesis 3a**: The positive implications of low residential mobility (one or two moves) with short-term duration of exposure to neighbourhood deprivation for educational attainment are stronger for children in intact families.
**Hypothesis 3b**: The negative implications of high residential mobility (three or more moves) for educational attainment are stronger for children in non-intact families.

### Social relations and the school context: The role of age and distance moved

In both residential mobility and neighbourhood effects research, social relations and the school context are considered important mechanisms influencing educational outcomes. Moving can disrupt a child’s peer network and school environment (Anderson et al., 2014), which is particularly harmful when experienced repeatedly as this requires frequent adjustments to a new environment (Mollborn et al., 2018). School mobility has indeed been found to negatively affect educational outcomes, especially when experienced frequently (Hansson and Gustafsson, 2020). Processes of socialisation and availability of quality schools are considered important ways in which the neighbourhood context affects educational outcomes (Nieuwenhuis et al., 2021). Moves to less deprived neighbourhoods might have positive implications for a child’s socialising context, such as positive role models, supportive peers and access to better quality schools. Those advantages may outweigh the negative impact of disrupted social networks and adjustment to a new school.

Regarding social relations and the school context, age and distance moved are key factors to consider. Moving and exposure to neighbourhood deprivation may have more severe consequences for educational attainment when experienced during key developmental stages (Coley and Kull, 2016). During the first years of their lives, children are more susceptible to changes in their immediate home environment and to family disruptions, whereas as children age social relations in the neighbourhood and school become more important (Coley and Kull, 2016). For educational attainment, it is therefore expected that residential moves and exposure to neighbourhood deprivation during the school-aged years, and especially adolescence, have a stronger impact due to the disruption of close peer relations and a possible change of school (Anderson et al., 2014). While short-distance moves may allow children to maintain social ties and remain in the same school, long-distance moves imply a more severe disruption of social ties and school context (Garboden et al., 2017). On the other hand, distant moves are more often planned and motivated by opportunities and preferences, potentially resulting in improvements to a child’s local environment which may be beneficial for educational trajectories (Hango, 2006). Nevertheless, taking into account family socio-economic background and exposure to neighbourhood deprivation, we could expect distant moves to have a stronger negative impact on educational attainment.

## The Dutch and Swedish context

Both the Netherlands and Sweden have witnessed shifts in their comprehensive social welfare system in the past decades characterised by increasingly liberal policies contributing to rising inequalities (Tammaru et al., 2015). Within this institutional context, the educational system plays a crucial role in the extent to which inequalities are transmitted. The Netherlands has a highly tracked educational system, while Sweden is known for its comprehensive educational system. International comparative studies suggest that educational inequalities are more severe in countries with a highly stratified educational system compared to countries with a comprehensive educational system (Griga and Hadjar, 2014). In the Netherlands, children are sorted into different levels of secondary education around the age of 12. Education is compulsory until the age of 16 and those not obtaining a basic qualification (see Measures section for details) are obliged to attend education until the age of 18. Sweden had nine years of compulsory education (‘grundskola’) until age 16 and three years of comprehensive upper-secondary education (‘gymnasium’) consisting of academic and vocational programmes. Upper-secondary education is not mandatory, but almost all Swedish youths enrol in such a programme. Despite these differences in educational systems, Andersson et al. (2023) find a similar negative association between contextual poverty and educational attainment for the Netherlands and Sweden.

The rise of liberal policies has impacted urban housing markets and contributed to an increase in income inequality and socio-economic segregation across European cities (Tammaru et al., 2015). Amsterdam and Stockholm differ in their housing market systems, urban policies and consequently levels of residential segregation. Socio-economic segregation is higher in Stockholm compared to Amsterdam, particularly in terms of segregation of poverty (Haandrikman et al., 2023). Geographically, deprived neighbourhoods in Amsterdam are located in the city’s post-war extensions in the West, North and Southeast and characterised by the absence of high-income residents rather than by a concentration of low-income residents. In Stockholm, deprived neighbourhoods are located in the Northwest and Southwest periphery extending beyond municipal boundaries and characterised by a strong overrepresentation of low-income residents.

Studying the consequences of childhood residential mobility and exposure to neighbourhood deprivation for educational attainment in these two capital cities can provide insights into similarities and differences in those processes in cities with different housing markets, levels of socio-economic segregation and educational systems. At the same time, studying both cities contributes to a more general understanding of residential mobility and neighbourhood effects among a highly mobile population.

## Data and methods

### Data and sample

I use longitudinal individual-level data from the administrative registers of Statistics Netherlands and Statistics Sweden, containing demographic, socio-economic and geographic information for the entire Dutch and Swedish population. Analyses were conducted for each country separately since the registers are only accessible in secure environments and cannot be linked. The metropolitan areas of Amsterdam and Stockholm are defined using the NUTS-3 classification (Eurostat, 2022) containing 13 municipalities for Amsterdam and 26 municipalities for Stockholm. The study population includes children born in 1995 in the most deprived neighbourhoods of the metropolitan areas; defined as the 20% lowest socio-economic status neighbourhoods (see Measures section for details). To analyse complete mobility trajectories, children who experienced an international migration, died, lived in institutional households, or had missing information on any of the variables were excluded. Additionally, one child per household was randomly selected. The final study population consists of 2248 children born in deprived neighbourhoods in Amsterdam metropolitan area and 3897 children born in deprived neighbourhoods in Stockholm metropolitan area. In Amsterdam those children predominantly reside within the city boundaries in the West, Southeast and North, in Stockholm the study population is more geographically dispersed across the metropolitan area, but with the majority residing at the edges of the city or neighbouring municipalities.

### Measures

All children were linked to their annual place of residence between birth and age 16. Residential addresses are based on coordinates of buildings in the Netherland, and coordinates of grid cells of 250 m * 250 m in urban areas and 1000 m * 1000 m in rural areas in Sweden. A move is defined as a change in residential address between two consecutive years and *moving frequency* is measured as the sum of moves between ages 0 and 16 categorised as: no move, low mobility (one or two moves), and high mobility (three or more moves). This categorisation was chosen based on prior research indicating a non-linear relation between childhood moves and outcomes, with negative effects found particularly for children moving three times or more (Mollborn et al., 2018).

To have a comparable measure of neighbourhood contexts in both cities and over time, individualised neighbourhoods were constructed for each childhood year using the *k*-nearest neighbour approach, which creates neighbourhoods based on a predetermined number of nearest neighbours for each individual (Andersson et al., 2023). This method is increasingly applied in international comparisons, as it does not rely on administrative boundaries (Haandrikman et al., 2023). I define neighbourhoods based on the 200 nearest neighbouring households with at least one member aged under 65. This small-scale measure was chosen because it more accurately reflects a child’s social environment, which is typically smaller in geographical scope, and because the association between contextual poverty and educational attainment has been found to be stronger at smaller scales (Andersson et al., 2023). Since the residential coordinates are more detailed for Amsterdam (buildings) compared to Stockholm (grid-cells), individualised neighbourhoods are larger in population in Stockholm with an average of 333 neighbouring households compared to 201 in Amsterdam. Individualised neighbourhoods are constructed using the nngeo package in R.

Neighbourhood socio-economic status was measured by aggregating individual and household-level socio-economic data to the individualised neighbourhoods (hereafter neighbourhoods). For each year, five neighbourhood variables were constructed: the proportion of residents aged 25–65 who are (1) employed, (2) receiving social benefits; and the proportion of households with at least one member aged 25–65 with (3) a low standardised household income (Q1), (4) a high standardised household income (Q5); and (5) the median standardised household income.^1^ Principal Component Analysis was performed for each year resulting in one factor of neighbourhood socio-economic status, categorised into quintiles (1 = very low to 5 = very high). *Exposure to neighbourhood deprivation* is measured as the number of childhood years lived in the most deprived neighbourhood (Q1), ranging from one year (at birth) to 17 years (entire childhood). Since all children in the study population were born in deprived neighbourhoods, the average duration of exposure to neighbourhood deprivation is relatively long (11 years). Therefore, in defining duration of exposure to neighbourhood deprivation, a relatively high cut-off point is used: short-term exposure is defined as having lived less than nine years in a deprived neighbourhood and long-term exposure as nine years or more. To assess the robustness of the results, additional analyses were conducted using an alternative cut-off of six years.

Combining the measure of frequency of moves and exposure to neighbourhood deprivation, children are categorised into five different *types of mobility patterns* (see Table 1): stayers with long exposure, low mobility short exposure, low mobility long exposure, high mobility short exposure and high mobility long exposure.^2^

**Table 1.** Prevalence of mobility categories (%) combining moving frequency and exposure to neighbourhood deprivation for Amsterdam (*N* = 2,248) and Stockholm (*N* = 3,897) metropolitan area.

Moving frequency	Exposure to neighbourhood deprivation			
	Short-term exposure		Long-term exposure	
	<9 years		≥9 years	
No moves	Excluded		Stayers	
			Amsterdam	16.7%
			Stockholm	16.4%
Low frequency 1–2 moves	Low mobility short exposure		Low mobility long exposure	
	Amsterdam	27.3%	Amsterdam	43.2%
	Stockholm	18.6%	Stockholm	36.7%
High frequency 3+ moves	High mobility short exposure		High mobility long exposure	
	Amsterdam	6.1%	Amsterdam	6.7%
	Stockholm	13.1%	Stockholm	15.2%

Sources: System of Social Statistical Datasets, Statistics Netherlands and Migrant Trajectories, Statistics Sweden (own calculations).

Propensity-score predictors were selected based on literature on indicators related to childhood residential mobility and educational outcomes. Migrant background is defined by having no foreign-born parents, or at least one foreign-born parent. Standardised household income is divided into quintiles (1 = very low to 5 = very high). Parental education is measured as the highest obtained educational level of either parent being low (compulsory), medium (upper-secondary), or high (post-secondary/tertiary).^3^ Parental employment is categorised as none or at least one parent employed. Parental union was measured as living with one or both parents at birth. Housing tenure is included as rental or owner-occupied housing.^4^ Since propensity-score predictors should be measured prior to moving, they are measured at birth when possible. For Sweden, all predictors are measured at birth, while for the Netherlands, due to data availability, employment is measured in 1999 and income in 2003, potentially after moving. Gender is included in all regression models. Additionally, robustness analyses are conducted among mobile children including the age at first move and average distance moved. Age at first move is categorised according to key school ages: ages 0–5 (pre-school); 6–12 (primary school); and 13–16 (secondary school). The average distance moved is categorised to reflect meaningful thresholds for potential school changes: less than 2 km; 2–10 km; and 10 km or more. In the final regression models, an interaction is included for family stability. This is measured throughout childhood and categorised as intact (living with both parents throughout childhood) or non-intact (living with a single parent or experiencing changes in the family structure such as parental separation or repartnering). In this model age at parental separation is additionally controlled for, categorised as: ages 0–5; 6–12; and 13–16.

The outcome studied is completion of secondary education, measured as having completed at least upper-secondary education by age 21. Given the differences in educational systems, the measure differs slightly between the countries but is indicative of access to the labour market or post-secondary education for both countries. For the Netherlands, it is measured as having obtained a basic qualification of at least an upper-secondary degree in vocational education (mbo-2), general secondary education (havo), or pre-university education (vwo). For Sweden, this is measured as having obtained a vocational or academic upper-secondary degree (gymnasium).

### Analytical strategy

Propensity-score methods aim at minimising selection bias by matching individuals on observed differences to create comparable groups and approximate randomisation. The use of propensity-score methods in studying childhood residential mobility to account for preexisting observed differences between mobile and non-mobile children has been shown to be fruitful (Garboden et al., 2017). In the current study, propensity-score weights are estimated for the five mobility types predicted by the described covariates. Propensity-score methods fit regression models to predict the probability (weights) of, in this case, a child experiencing a certain type of mobility based on the assigned covariates. Those weights are subsequently used in the analyses to minimise differences between the mobility groups. Given that multiple groups of movers are compared, propensity score weights are estimated using Generalised Boosted Models for multiple treatments fitting a multinomial propensity score (McCaffrey et al., 2013). This approach compares each of the mobility categories using multiple regression trees to optimise covariate balance. Covariate balance was assessed, indicating that the mobility groups were well balanced on all included covariates (see Appendix A for the unweighted and weighted means of all covariates for the mobility groups and the unweighted and weighted absolute standardised differences).

Three logistic regression models are used to analyse the association between the five different mobility types and completion of secondary education.^5^ Model 1 analyses the baseline association using an unweighted logistic regression model controlling for gender. In model 2 the estimated propensity-weights and covariates (migrant background, household income, parental employment, parental educational attainment, family structure and housing tenure at birth) are added to account for differences in the probability of experiencing the different mobility types. To assess the robustness of the findings, models 1 and 2 are repeated using an alternative cut-off for exposure to neighbourhood deprivation as well as for mobile children, additionally including age at first move and average distance moved. Model 3 adds an interaction between family stability throughout childhood and mobility type controlling additionally for age at parental separation. All models were conducted separately for Amsterdam and Stockholm. For Amsterdam, the high mobility groups were merged in model 3 given the small number of children experiencing frequent moving.

## Results

### Childhood residential mobility patterns and selective mobility

Residential mobility is very common among children born in deprived neighbourhoods in Amsterdam and Stockholm, with 84% moving at least once in childhood. The majority of children move once or twice, and frequent moving is more common among children born in Stockholm (28%) compared to Amsterdam (13%).

Table 2 shows descriptive statistics of mobility indicators (age, distance, frequency), exposure to neighbourhood deprivation and characteristics at birth by type of mobility trajectory for children born in deprived neighbourhoods compared to all children of the same birth cohort born in the metropolitan areas. The majority of mobile children make their first move before the age of five. Children with low mobility and long exposure make their first move later in childhood, while highly mobile children are on average younger. Moves during primary school ages (6–12) are common across all groups, but particularly high among highly mobile children. Moves between ages 13 and 16 are only common among highly mobile children. Children moving frequently with short-term exposure to deprivation move on average the longest distances, while children with low mobility and long-term exposure to deprivation on average move mostly nearby. Across all types of mobility, the average moving frequency is slightly higher among children born in Stockholm compared to Amsterdam. In both cities, children from deprived neighbourhoods on average live for around 11 years in a deprived neighbourhood. Children with short exposure to neighbourhood deprivation mainly experience this exposure during early childhood (age 0–5) and live on average less than a year in deprived areas after the age of six.

**Table 2.** Mobility indicators, exposure to neighbourhood deprivation and characteristics at birth for children born in deprived neighbourhoods by type of mobility compared to all children of the same birth cohort born in Amsterdam and Stockholm metropolitan area.

	Born in deprived neighbourhood					Metropolitan area average
	Stayers	Low mobility		High mobility		
		Short exposure	Long exposure	Short exposure	Long exposure	
	Mean (SD)	Mean (SD)	Mean (SD)	Mean (SD)	Mean (SD)	Mean (SD)
	Amsterdam metropolitan area					
Mobility and Neighbourhood Indicators						
Age first move		3.80	6.65	2.28	3.03	5.05
		(2.77)	(4.57)	(1.50)	(2.66)	(3.97)
0–5		0.75	0.48	0.95	0.85	0.44
6–12		0.24	0.39	0.05	0.15	0.20
13–16		0.01	0.13	0.00	0.00	0.05
Moved age 6–12		0.43	0.52	0.87	0.89	0.34
Moved age 13–16		0.09	0.23	0.55	0.55	0.11
Average distance moved		12.11	5.45	18.17	13.06	8.18
		(14.64)	(10.31)	(18.29)	(18.84)	(17.26)
<2 km		0.21	0.46	0.04	0.25	0.23
2–10 km		0.37	0.41	0.36	0.39	0.24
>10 km		0.42	0.14	0.59	0.36	0.22
Number of moves		1.36	1.29	3.76	3.56	1.13
		(0.48)	(0.45)	(0.96)	(0.99)	(1.11)
Years of exposure to neighbourhood deprivation	16.45	4.05	14.81	4.09	13.67	3.40
	(1.51)	(2.36)	(2.69)	(2.30)	(2.60)	(5.70)
Years of exposure age 0–5	5.81	3.29	5.62	2.95	5.22	1.20
	(0.73)	(1.82)	(1.01)	(1.73)	(1.25)	(2.15)
Years of exposure age 6–12	6.81	0.49	6.17	0.78	5.69	1.40
	(0.69)	(0.97)	(1.38)	(1.38)	(1.66)	(2.56)
Years of exposure age 13–16	3.84	0.27	3.02	0.36	2.76	0.80
	(0.67)	(0.83)	(1.57)	(0.79)	(1.57)	(1.52)
Characteristics at birth						
Parental migrant background						
No migrant parents	0.15	0.40	0.12	0.47	0.19	0.62
At least one migrant parent	0.85	0.60	0.88	0.53	0.81	0.38
Standardised household income quintiles						
Q1	0.54	0.30	0.55	0.38	0.51	0.24
Q2	0.28	0.25	0.25	0.28	0.27	0.20
Q3	0.11	0.24	0.14	0.19	0.15	0.21
Q4	0.07	0.14	0.05	0.10	0.07	0.19
Q5	0.02	0.06	0.01	0.05	0.01	0.17
Parental employment						
Neither parent employed	0.46	0.22	0.46	0.37	0.45	0.17
At least one parent employed	0.54	0.78	0.54	0.63	0.55	0.83
Highest parental education						
Low	0.51	0.22	0.43	0.22	0.39	0.17
Medium	0.18	0.29	0.30	0.41	0.41	0.28
High	0.07	0.26	0.08	0.18	0.09	0.30
Unknown	0.25	0.22	0.19	0.19	0.12	0.25
Parental union						
Both parents	0.81	0.73	0.70	0.59	0.58	0.84
Single parent	0.19	0.27	0.30	0.41	0.42	0.16
Housing tenure						
Rental	0.98	0.97	0.99	0.98	1.00	0.63
Home-owner	0.02	0.03	0.01	0.02	0.00	0.37
*N*	376	613	971	138	150	11,353
			Stockholm metropolitan area			
Mobility and Neighbourhood Indicators						
Age first move		3.49	6.68	2.11	2.93	4.51
		(2.69)	(4.57)	(1.58)	(2.42)	(3.85)
0–5		0.81	0.48	0.96	0.85	0.53
6–12		0.18	0.38	0.05	0.15	0.18
13–16		0.01	0.14	0.00	0.00	0.05
Moved age 6–12		0.38	0.53	0.86	0.88	0.42
Moved age 13–16		0.13	0.28	0.56	0.61	0.19
Average distance moved		23.70	13.08	34.95	33.49	20.92
		(68.76)	(50.39)	(59.51)	(59.21)	(63.14)
<2 km		0.27	0.54	0.14	0.19	0.25
2–10 km		0.45	0.31	0.38	0.38	0.28
>10 km		0.28	0.15	0.49	0.43	0.23
Number of moves		1.47	1.39	4.07	4.08	1.63
		(0.50)	(0.49)	(1.35)	(1.47)	(1.56)
Years of exposure to neighbourhood deprivation	16.61	3.87	14.74	4.19	13.49	3.40
	(1.31)	(2.34)	(2.79)	(2.45)	(2.76)	(5.58)
Years of exposure age 0–5	5.85	3.06	5.75	2.86	5.31	1.20
	(0.64)	(1.73)	(0.78)	(1.69)	(1.16)	(2.14)
Years of exposure age 6–12	6.87	0.51	6.15	0.84	5.52	1.40
	(0.56)	(0.95)	(1.44)	(1.26)	(1.74)	(2.49)
Years of exposure age 13–16	3.89	0.30	2.85	0.48	2.66	0.80
	(0.51)	(0.86)	(1.63)	(1.03)	(1.59)	(1.48)
Characteristics at birth						
Parental migrant background						
No migrant parents	0.18	0.53	0.17	0.60	0.33	0.70
At least one migrant parent	0.82	0.47	0.83	0.40	0.68	0.30
Standardised household income quintiles						
Q1	0.77	0.38	0.77	0.51	0.76	0.32
Q2	0.14	0.29	0.16	0.27	0.15	0.21
Q3	0.06	0.18	0.05	0.16	0.07	0.19
Q4	0.02	0.11	0.02	0.05	0.02	0.16
Q5	0.01	0.03	0.01	0.01	0.00	0.13
Parental employment						
Neither parent employed	0.32	0.08	0.31	0.14	0.35	0.09
At least one parent employed	0.68	0.92	0.69	0.86	0.65	0.91
Highest parental education						
Low	0.25	0.06	0.18	0.10	0.20	0.06
Medium	0.51	0.52	0.51	0.55	0.54	0.46
High	0.24	0.42	0.32	0.36	0.26	0.48
Parental union						
Both parents	0.85	0.90	0.84	0.81	0.74	0.88
Single parent	0.15	0.10	0.16	0.19	0.26	0.12
Housing tenure						
Rental	0.88	0.80	0.91	0.86	0.92	0.46
Home-owner	0.12	0.20	0.09	0.14	0.08	0.54
*N*	638	725	1,430	510	594	19,607

Sources: System of Social Statistical Datasets, Statistics Netherlands and Migrant Trajectories, Statistics Sweden (own calculations).

As expected, children born in deprived neighbourhoods are generally in a more disadvantaged situation compared to the total population born in the metropolitan areas, which applies to stayers and all types of movers. Among stayers and children moving with long-term exposure to neighbourhood deprivation a higher proportion has a migrant background and the socio-economically disadvantaged family situation is more pronounced. Children moving with short-term exposure to neighbourhood deprivation less often have migrant parents and are more often in socio-economically advantaged family situations at birth compared to their peers born in a similar neighbourhood. Particularly children with low mobility and short-term exposure are socio-economically more advantaged and fairly similar to peers born in more affluent neighbourhoods. Children living in single-parent households are overrepresented among frequent movers, in Stockholm particularly in combination with long-term exposure to neighbourhood deprivation. The proportion of children in owner-occupied housing at birth is very low among children born in deprived neighbourhoods, but somewhat higher among those with short-term exposure to deprivation, although differences are small.

### Completion of secondary education

The disadvantaged situation of children born in deprived neighbourhoods is also reflected in their completion of secondary education. Among children born in deprived neighbourhoods a lower proportion completed secondary education by the age of 21 (Amsterdam: 75%; Stockholm: 81%) compared to all children born in the metropolitan area (Amsterdam: 82%; Stockholm: 88%). Figure 1 shows the results of the unweighted baseline logistic regression models (model 1) and the propensity-weighted logistic regression models (model 2) and displays the odds ratios for completing secondary education for the different mobility types for both Amsterdam and Stockholm (see Appendix Table B1 for the full models).

**Figure 1.**
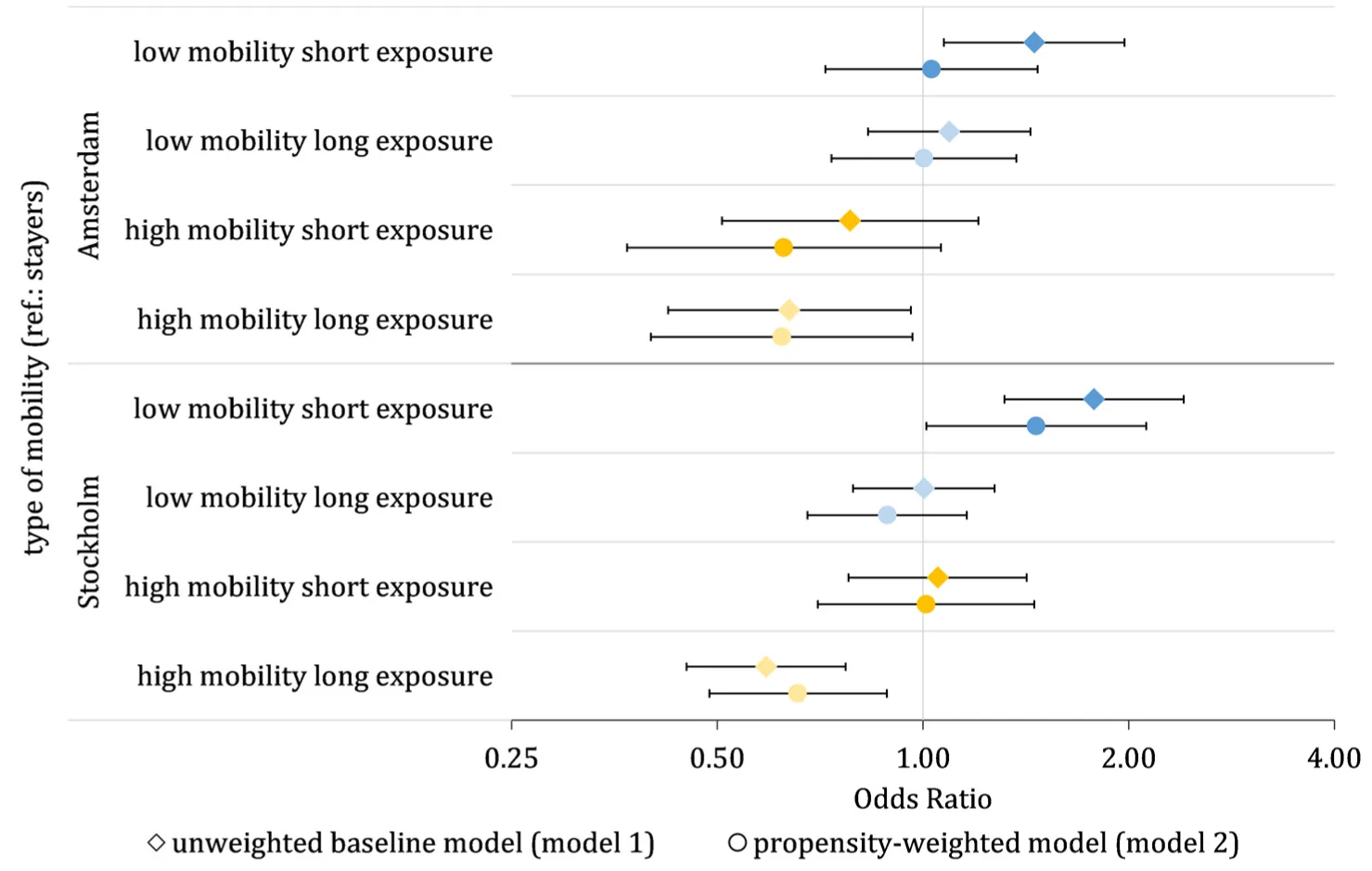
Odds ratios unweighted baseline logistic regression (Model 1) and propensity-weighted logistic regression (Model 2) for completion of secondary education by childhood mobility type (ref. = stayers) for children born in deprived neighbourhoods in Amsterdam and Stockholm metropolitan area. Note: Propensity-weighted model includes propensity-weights based on migrant background, household income, parental employment, parental educational attainment, family structure and housing tenure at birth and abovementioned covariates as well as gender. See Appendix Table B1 for the full models. Data sources: System of Social Statistical Datasets, Statistics Netherlands and Migrant Trajectories, Statistics Sweden (own calculations).

For both metropolitan areas, the unweighted baseline model shows that children with low mobility and short-term exposure to neighbourhood deprivation have higher odds of completing secondary education compared to stayers (Amsterdam: OR: 1.455, *p* = 0.016; Stockholm: OR: 1.780, *p* < 0.001). For Amsterdam, in the propensity-weighted model this association is reduced to non-significance, indicating that the difference can mainly be explained by selective upward neighbourhood mobility. For Stockholm, this association is reduced but remains significant (OR: 1.464, *p* = 0.044), indicating a beneficial association for moving once or twice towards better neighbourhoods even after accounting for selective mobility patterns.

In both metropolitan areas, children with high mobility and long-term exposure have significantly lower odds of completing secondary education compared to stayers, both in the baseline model (Amsterdam: OR: 0.638, *p* = 0.031; Stockholm: OR: 0.590, *p* < 0.001) as well as in the propensity-weighted model (Amsterdam: OR: 0.622, *p* = 0.034; Stockholm: OR: 0.656, *p* = 0.006). This indicates that while frequent movers are indeed a group of children in a more disadvantaged situation, there is an additional impact of moving frequently in combination with long-term exposure to neighbourhood deprivation on the likelihood of completing secondary education. No difference is found in the completion of secondary education between children with low mobility and long-term exposure or high mobility and short-term exposure to neighbourhood deprivation compared to stayers.

### Heterogeneity by family stability

To assess heterogeneity in the associations, a propensity-weighted logistic regression (model 3) was conducted including an interaction term for type of mobility and family stability controlling for age at parental separation. Table 3 shows the proportion of children in each of the mobility groups by family stability. Mobility is higher among children in non-intact families compared to children in intact families. More importantly, the type of mobility clearly differs: frequent moving is much more common among children in non-intact families, while children in intact families more often experience low mobility with shorter exposure to neighbourhood deprivation.

**Table 3.** Type of mobility by family stability for children born in deprived neighbourhoods in Amsterdam and Stockholm metropolitan area (%).

Type of mobility	Amsterdam		Stockholm	
	Intact family	Non-intact family	Intact family	Non-intact family
Stayers	21	12	19	14
Low mobility short exposure	31	24	26	12
Low mobility long exposure	43	44	40	34
High mobility short exposure	2	10	8	17
High mobility long exposure	3	10	7	22
Total (*N*)	1,111	1,137	1,822	2,075

Data sources: System of Social Statistical Datasets, Statistics Netherlands and Migrant Trajectories, Statistics Sweden (own calculations).

Figure 2 shows the predicted probabilities of completing secondary education for the different mobility types of children living with both parents throughout childhood and those growing up in non-intact families (see Appendix Table B2 for the full model). For Amsterdam, there is no significant difference between children in intact or non-intact families. Although frequent moving seems somewhat more detrimental for children in non-intact families, the difference is not significant, partly because repeated mobility is rare among children living with both parents throughout childhood. For Stockholm, significant differences were found. Children living with both parents throughout childhood with low mobility and short-term exposure to neighbourhood deprivation are significantly more likely to complete secondary education compared to stayers living with both parents, while this does not apply to children in non-intact families. Conversely, high mobility and long-term exposure to neighbourhood deprivation is more detrimental for children in non-intact families, who are significantly less likely to complete secondary education compared to stayers, while this does not apply to children in intact families. A clear gap thus arises: children living with both parents who move once or twice to less deprived neighbourhoods have a 93% probability of completing secondary education, while children in non-intact families moving frequently with long-term exposure to neighbourhood deprivation have a 70% probability. While this gap is also observed in Amsterdam, the difference is smaller and non-significant: children in intact families with low mobility and decreased exposure have a 77% probability to complete secondary education and this is 68% for children in non-intact families with high mobility. Although caution is warranted due to the small proportion of children in intact families experiencing high mobility, these results suggest that family stability may play a more limited role in explaining differences in educational attainment across mobility types in Amsterdam compared to Stockholm.

**Figure 2.**
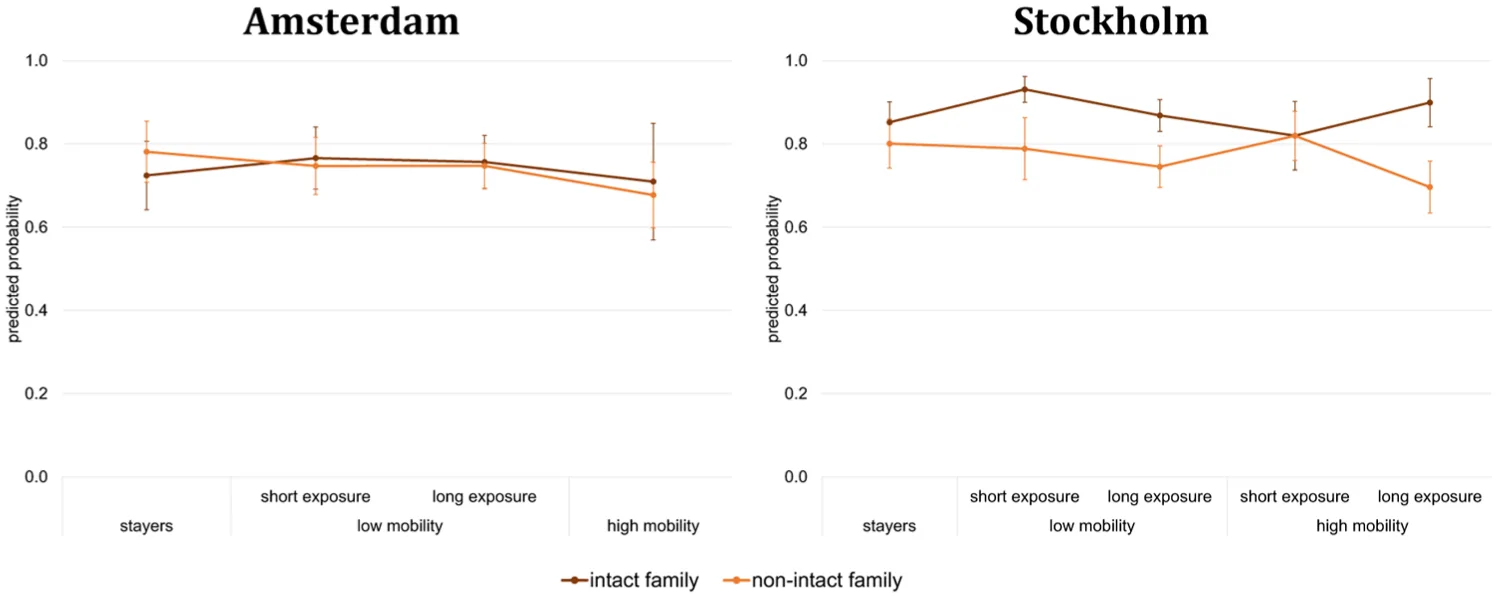
Predicted probabilities propensity-weighted logistic regression completion of secondary education interaction childhood mobility and family stability (Model 3) of children born in deprived neighbourhoods in Amsterdam and Stockholm metropolitan area. Note: Model includes propensity-weights based on migrant background, household income, parental employment, parental educational attainment, family structure and housing tenure at birth and abovementioned covariates as well as gender and age at parental separation. See Appendix Table B2 for the full models. Data sources: System of Social Statistical Datasets, Statistics Netherlands and Migrant Trajectories, Statistics Sweden (own calculations).

### Robustness checks: Duration of exposure, age and distance moved

Given the importance of duration of exposure to neighbourhood deprivation (Chetty and Hendren, 2018), additional analyses were conducted using an alternative cut-off of six years for exposure to neighbourhood deprivation. Those analyses show similar results (see Appendix Table C1). The positive association for children with low mobility and short-term exposure is somewhat stronger in these models, confirming the importance of exposure time.

Age of moving and distance moved are important factors to consider in relation to the disruption of peer relations and the school context (Garboden et al., 2017). Therefore, additional analyses were conducted accounting for age at first move and distance moved among mobile children (Amsterdam: *N* = 1872; Stockholm: *N* = 3259; Appendix Table C2). Results show that age at first move is not significantly associated with completion of secondary education. Moving on average more than 10 km is associated with a lower likelihood to complete secondary education. Importantly, results for the different mobility types are robust when accounting for age at first move and distance moved, suggesting that the found associations between mobility types and educational attainment are not attributable to these factors, which serve as proxies for school-related changes.

## Conclusion and discussion

Focussing on children born in deprived neighbourhoods in Amsterdam and Stockholm, this study has demonstrated that residential mobility and exposure to neighbourhood deprivation play an important role in accumulations of (dis)advantage over a child’s life course. It shows that a combination of negative or positive processes in a child’s life related to residential mobility, neighbourhood deprivation and family stability contribute to disparities in educational attainment. Three general conclusions can be drawn from the findings.

First, the type of residential mobility and neighbourhood exposure experienced by children born in deprived areas is highly selective. In line with previous studies (Coulton et al., 2012; Gambaro et al., 2017; Goldsmith et al., 2017), the type of mobility is found to be related to migrant background, family socio-economic background, family structure and housing tenure. Children with non-migrant backgrounds and in more advantaged socio-economic family situations more often experience moves resulting in a reduced exposure to neighbourhood deprivation. Children with migrant parents and in more disadvantaged socio-economic family situations more often stay or move within deprived areas. Frequent moving is most common among children in single-parent families, while children living with both parents more often stay or move towards less deprived neighbourhoods. These results point to potential accumulations across different life domains, in which stable families with resources are moving to better neighbourhoods, while less stable families with a lack of resources are moving frequently and move within or between deprived neighbourhoods. It furthermore underlines the importance of accounting for selective mobility patterns.

Second, this study finds partial support for the upward mobility hypothesis (Hypothesis 1) that moving out of a deprived neighbourhood with a reduced exposure to neighbourhood deprivation would be beneficial for educational attainment. Results show that upward mobility – when mobility is not experienced repeatedly – is associated with a higher likelihood of completing secondary education, but that this is completely (Amsterdam) and partially (Stockholm) explained by selective upward mobility of more affluent families. Confirming Hypothesis 3a, this positive association is stronger for children living with both parents throughout childhood. This has important implications for how we study neighbourhood effects, as it points not only to more advantaged stable families being able to move out of deprivation but also to an accumulation of a positive family and neighbourhood environment allowing children to have a successful educational career.

Third, the study points to a detrimental impact of frequent moving for completion of secondary education, in line with expectations and previous studies (Haugan and Myhr, 2019; Tønnessen et al., 2016). I expected the negative association for frequent moving to be present in combination with both short- and long-term exposure to neighbourhood deprivation but to be more salient in combination with long-term exposure (Hypothesis 2). However, in both metropolitan areas, frequent moving is only significantly associated with a decreased likelihood of completing secondary education when accompanied with long-term exposure to neighbourhood deprivation, even after accounting for preexisting differences. For Amsterdam, however, those results should be interpreted with some caution as only a small proportion of children experience frequent moves and additional analyses including two cohorts of children do not confirm the results. In line with expectations (Hypothesis 3b) and previous studies (Haugan and Myhr, 2019; Tucker et al., 1998), the strongest negative impact of frequent moving with long-term exposure to neighbourhood deprivation is found for children in non-intact families, particularly in Stockholm. Those findings point to a pattern of cumulative disadvantage of frequent residential and family disruptions, in which children repeatedly need to adjust to changing residential environments and family situations.

The direction of most of the associations found is similar in Amsterdam and Stockholm, suggesting a more general pattern in the combined role of residential mobility and exposure to neighbourhood deprivation for education. However, results also point to differences. Overall, the combined influence of positive (low residential mobility with short exposure to neighbourhood deprivation) or negative (high mobility with long exposure) childhood circumstances on educational attainment is more pronounced in Stockholm. This is also confirmed by the robustness analyses including an interaction term for residential mobility and exposure to neighbourhood deprivation. Most importantly, after accounting for selective mobility patterns, no significant association is found for upward neighbourhood mobility in Amsterdam. Furthermore, in both metropolitan areas, frequent residential mobility combined with long-term exposure to neighbourhood deprivation is negatively associated with educational attainment. However, the difference between frequent residential mobility with short- or long-term exposure is not visible in Amsterdam, suggesting that repeated mobility itself plays a more important role rather than a combined impact with neighbourhood exposure. Finally, family stability appears to play a less significant role in Amsterdam than in Stockholm in the association between residential mobility, exposure to neighbourhood deprivation and educational attainment.

Although a comprehensive comparison between the metropolitan areas was not possible given the different data sources and contextual differences, three possible explanations can be considered. First, the effects of neighbourhood context on educational outcomes tend to be stronger in countries with higher levels of segregation and more pronounced concentrations of poverty. The higher levels of socio-economic segregation and concentrated poverty in Stockholm compared to Amsterdam (Haandrikman et al., 2023) may therefore help explain the stronger impact of moving out of a deprived area. As individualised neighbourhood are increasingly used in international comparative studies, this study underlines the importance of this approach to capture differences in consequences of exposure to neighbourhood deprivation across cities. Second, differences in the educational system between the two countries may play a role. In the Netherlands, children are sorted into educational levels at a young age. At this age, children might not yet have benefited from moving to a more affluent neighbourhood as this impact might not be apparent until later in childhood, after a period of coping and adjusting to a new environment (Chetty et al., 2016; Hango, 2006). Third, frequent residential mobility and parental separation are more common in Stockholm than in Amsterdam. This not only provides greater variation for assessing the role of family stability among frequent movers, but may also help explain why family stability plays a more important role in the context of Stockholm. Additionally, limitations in the Dutch data, in which it was not possible to measure household income and parental employment at birth, mean that the results for Amsterdam should be interpreted with some caution. Nevertheless, future research should pay more attention to how contextual differences in residential segregation, educational systems, and family arrangements shape long-term consequences of residential mobility and exposure to neighbourhood deprivation.

The findings of this study underline the need for more attention in literature to the diversity of children’s moving experiences and circumstances and the interrelated impact of residential mobility and neighbourhood exposure on educational outcomes. First, findings underline the importance of addressing selection bias when studying residential mobility and neighbourhood effects. This study aimed at minimising selection bias by studying a comparable group of children focussing on those born in deprived neighbourhoods and correcting for differences in observed characteristics at birth using propensity-score methods. Nevertheless, causal claims cannot be made due to potential unobserved characteristics and co-occurring events that might confound or mediate the found associations (Garboden et al., 2017). Sibling designs appear promising to address this bias when studying a larger group of children (see e.g., Chetty and Hendren, 2018; Tarkiainen et al., 2024).

Second, more research is needed to explore the mechanisms underlying the found associations. With the current study pointing to accumulations of (dis)advantage across life domains, future studies should examine the interrelatedness of socialisation contexts and how they may exacerbate or mitigate disruptions and disadvantage. Changes in the school context especially may play an important role, as school mobility can be disruptive but may also mitigate negative effects depending on the school quality after moving. This is especially relevant in light of the finding that distant moves appear more detrimental for educational outcomes. Additionally, disruptions in the family context in relation to residential moves are important to explore in more detail as findings suggest that non-intact families are overrepresented in patterns of frequent residential mobility.

Finally, while this study points to variation between types of mobility, there might also be diversity within mobility types. Most importantly, a reduction in exposure to neighbourhood deprivation might indicate a move to a slightly less deprived neighbourhood for some and to a much-improved neighbourhood for others. Additionally, the age of moving might vary within mobility types. Descriptive results for children with low mobility and short-term exposure point to an exit out of deprived areas in early childhood. Although results are robust to an alternative cut-off for duration of exposure to neighbourhood deprivation and when accounting for age at first move and distance moved among mobile children, future research could add to our understanding by studying the impact of upward mobility at different ages.

In sum, the applied multidimensional and longitudinal perspective advances our understanding of contextual accumulations of (dis)advantage over a child’s life course. It points to a pattern in which residential moves out of deprived areas improve the situation of children in an already more stable and advantaged situation while frequent moving within deprived areas detriments the educational attainment of children in an already less stable and disadvantaged situation. With this, it underscores that combinations of positive or negative childhood circumstances shape educational disparities. Scholars should attempt to further integrate literature on residential mobility and neighbourhood effects to unpack diversity in childhood experiences that may have long-term consequences for inequalities of opportunities.

## Figures and Tables

**Figure A1.**
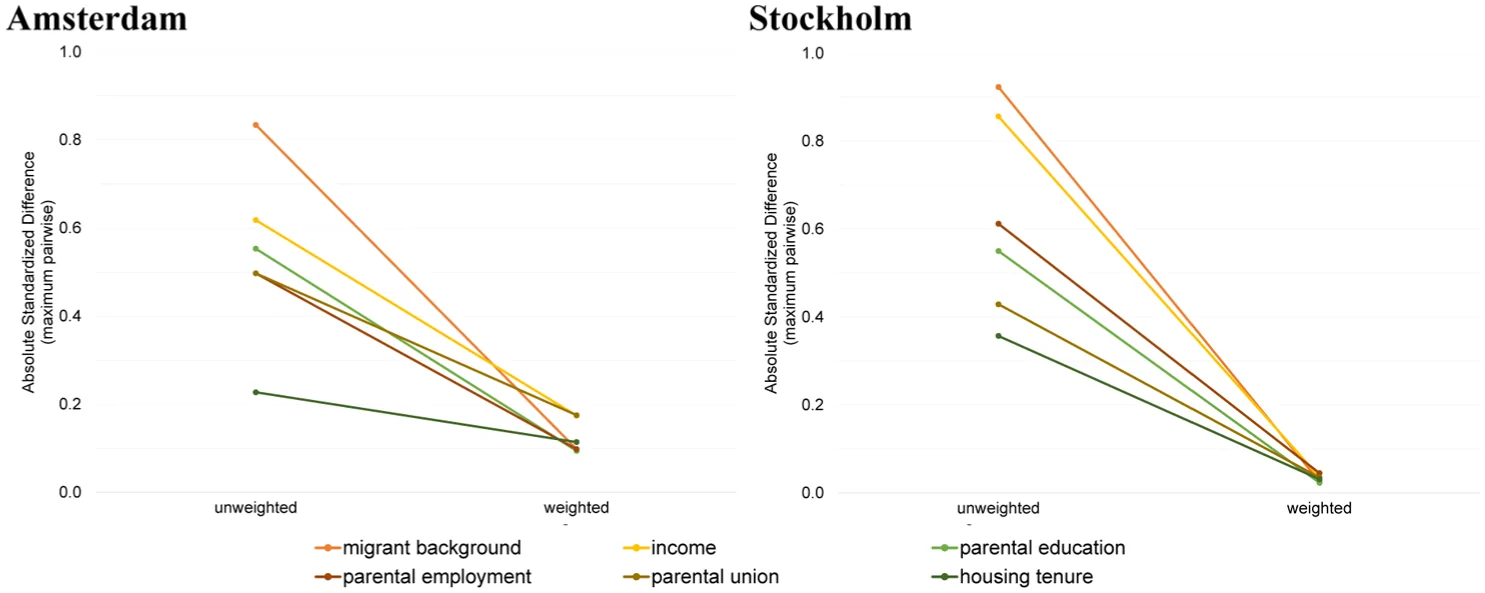
Absolute standardized difference on covariates before and after weighting on all covariates for Amsterdam and Stockholm metropolitan area. Sources: System of Social Statistical Datasets, Statistics Netherlands and Migrant Trajectories, Statistics Sweden (own calculations).

**Figure C1.**
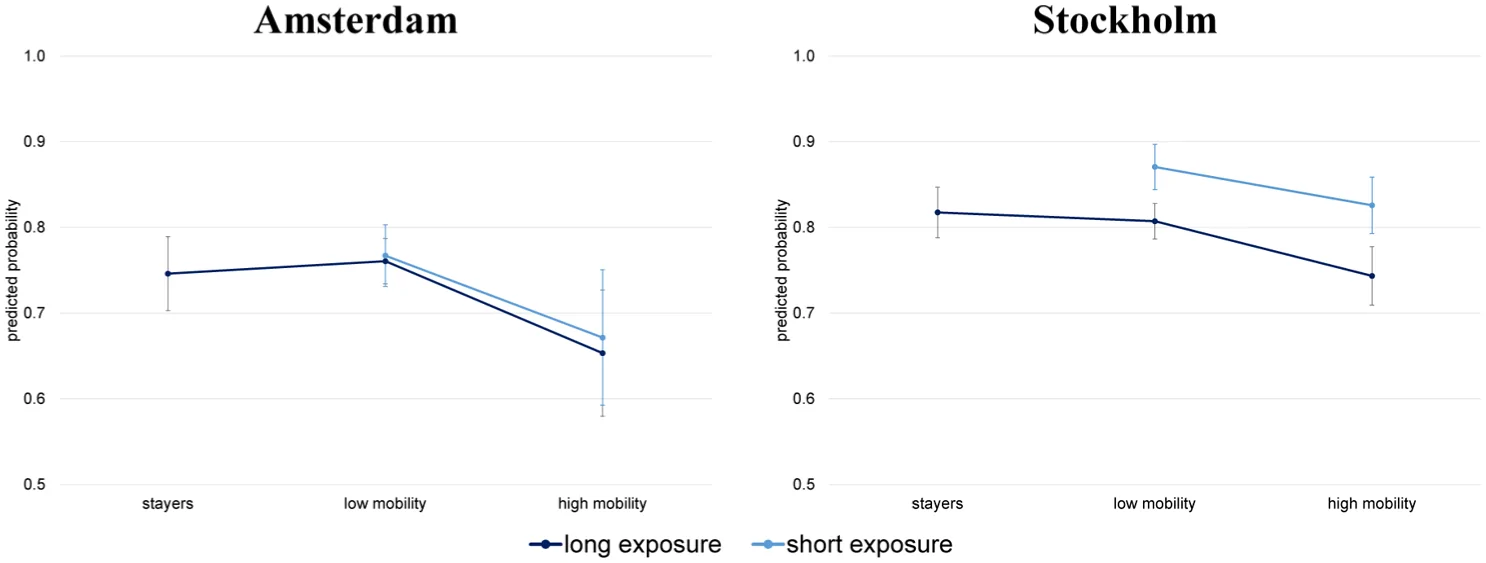
Predicted probabilities unweighted logistic regression completion of secondary education interaction childhood residential mobility and exposure to neighbourhood deprivation of children born in deprived neighbourhoods in metropolitan areas of Amsterdam and Stockholm. Note: Models include covariates gender, migrant background, household income, parental employment, parental educational attainment, family structure and housing tenure at birth. Data sources: System of Social Statistical Datasets, Statistics Netherlands and Migrant Trajectories, Statistics Sweden (own calculations).

## Appendix A. Balance covariates

**Table A1.** Unweighted and weighted means of covariates at birth by mobility type for children born in deprived neighbourhoods in Amsterdam metropolitan area (*N* = 2,248).

Covariates	Stayers	Low mobility		High mobility		Stayers	Low mobility		High mobility	
		Short exposure	Long exposure	Short exposure	Long exposure		Short exposure	Long exposure	Short exposure	Long exposure
	Unweighted mean					Weighted mean				
Parental migrant background										
No migrant parents	0.15	0.40	0.12	0.47	0.19	0.22	0.23	0.22	0.23	0.19
At least one migrant parent	0.85	0.60	0.88	0.53	0.81	0.79	0.77	0.78	0.77	0.81
Standardised household income quintiles										
Q1	0.54	0.30	0.55	0.38	0.51	0.47	0.47	0.48	0.42	0.50
Q2	0.28	0.25	0.25	0.28	0.27	0.27	0.26	0.26	0.28	0.27
Q3	0.11	0.24	0.14	0.19	0.15	0.16	0.16	0.16	0.19	0.15
Q4	0.07	0.14	0.05	0.10	0.07	0.08	0.08	0.08	0.08	0.07
Q5	0.02	0.06	0.01	0.05	0.01	0.02	0.03	0.02	0.04	0.02
Parental employment										
Neither parent employed	0.46	0.22	0.46	0.37	0.45	0.39	0.39	0.40	0.38	0.43
At least one parent employed	0.54	0.78	0.54	0.63	0.55	0.61	0.61	0.60	0.62	0.58
Highest parental education										
Low	0.51	0.22	0.43	0.22	0.39	0.38	0.37	0.38	0.30	0.39
Medium	0.18	0.29	0.30	0.41	0.41	0.28	0.29	0.29	0.31	0.38
High	0.07	0.26	0.08	0.18	0.09	0.14	0.14	0.13	0.15	0.10
Unknown	0.25	0.22	0.19	0.19	0.12	0.20	0.20	0.20	0.24	0.14
Parental union										
Both parents	0.81	0.73	0.70	0.59	0.58	0.73	0.71	0.71	0.68	0.65
Single parent	0.19	0.27	0.30	0.41	0.42	0.27	0.29	0.29	0.33	0.35
Housing tenure										
Rental	0.98	0.97	0.99	0.98	1.00	0.99	0.99	0.99	0.99	1.00
Home-owner	0.02	0.03	0.01	0.02	0.00	0.01	0.01	0.01	0.01	0.00

Data source: System of Social Statistical Datasets, Statistics Netherlands (own calculations).

**Table A2.** Unweighted and weighted means of covariates at birth by mobility type for children born in deprived neighbourhoods in Stockholm metropolitan area (*N* = 3,897).

Covariates	Stayers	Low mobility		High mobility		Stayers	Low mobility		High mobility	
		Short exposure	Long exposure	Short exposure	Long exposure		Short exposure	Long exposure	Short exposure	Long exposure
	Unweighted mean					Weighted mean				
Parental migrant background										
No migrant parents	0.18	0.53	0.17	0.60	0.33	0.31	0.32	0.32	0.33	0.31
At least one migrant parent	0.82	0.47	0.83	0.40	0.68	0.69	0.68	0.69	0.68	0.69
Standardised household income quintiles										
Q1	0.77	0.38	0.77	0.51	0.76	0.66	0.66	0.67	0.65	0.67
Q2	0.14	0.29	0.16	0.27	0.15	0.19	0.19	0.19	0.21	0.19
Q3	0.06	0.18	0.05	0.16	0.07	0.10	0.10	0.09	0.10	0.09
Q4	0.02	0.11	0.02	0.05	0.02	0.04	0.04	0.04	0.04	0.04
Q5	0.01	0.03	0.01	0.01	0.00	0.01	0.01	0.01	0.01	0.01
Parental employment										
Neither parent employed	0.32	0.08	0.31	0.14	0.35	0.25	0.24	0.25	0.26	0.26
At least one parent employed	0.68	0.92	0.69	0.86	0.65	0.75	0.76	0.75	0.74	0.74
Highest parental education										
Low	0.25	0.06	0.18	0.10	0.20	0.16	0.16	0.16	0.15	0.16
Medium	0.51	0.52	0.51	0.55	0.54	0.52	0.52	0.52	0.52	0.52
High	0.24	0.42	0.32	0.36	0.26	0.32	0.32	0.32	0.33	0.32
Parental union										
Both parents	0.85	0.90	0.84	0.81	0.74	0.84	0.84	0.83	0.83	0.83
Single parent	0.15	0.10	0.16	0.19	0.26	0.16	0.16	0.17	0.17	0.17
Housing tenure										
Rental	0.88	0.80	0.91	0.86	0.92	0.88	0.88	0.88	0.88	0.89
Home-owner	0.12	0.20	0.09	0.14	0.08	0.12	0.12	0.12	0.13	0.12

Data source: Migrant Trajectories, Statistics Sweden (own calculations).

## Appendix B. Full tables weighted logistic regression models

**Table B1.** Odds ratios (OR) unweighted baseline logistic regression (Model 1) and propensity-weighted logistic regression (Model 2) completion of secondary education age 21 for children born in deprived neighbourhoods in Amsterdam and Stockholm metropolitan area.

Variables	Amsterdam			Stockholm		
	OR	95% CI	*p*	OR	95% CI	*p*
	Model 1: Unweighted baseline model					
Type of mobility (ref. = stayers)						
Low mobility short exposure	1.455	1.07–1.97	0.016	1.780	1.32–2.41	<0.001
Low mobility long exposure	1.093	0.83–1.44	0.526	1.003	0.79–1.27	0.983
High mobility short exposure	0.782	0.51–1.21	0.265	1.051	0.78–1.42	0.747
High mobility long exposure	0.638	0.42–0.96	0.031	0.590	0.45–0.77	<0.001
Gender (ref. = male)						
Female	2.047	1.68–2.50	<0.001	1.365	1.16–1.61	<0.001
	Model 2: Propensity-weighted model					
Type of mobility (ref. = stayers)						
Low mobility short exposure	1.029	0.72–1.47	0.875	1.464	1.01–2.12	0.044
Low mobility long exposure	1.003	0.74–1.37	0.983	0.887	0.68–1.16	0.379
High mobility short exposure	0.626	0.37–1.06	0.082	1.011	0.70–1.46	0.953
High mobility long exposure	0.622	0.40–0.97	0.034	0.656	0.49–0.89	0.006
Sex (ref. = male)						
Female	1.847	1.39–2.46	<0.001	1.329	1.08–1.63	0.006
Parental migrant background (ref. = no migrant parent)						
At least one migrant parent	0.945	0.69–1.30	0.726	1.602	1.27–2.02	<0.001
Standardised household income quintiles (ref. = Q1)						
Q2	1.110	0.78–1.58	0.563	1.170	0.89–1.54	0.261
Q3	1.133	0.73–1.77	0.582	1.424	0.95–2.13	0.085
Q4	0.962	0.53–1.73	0.898	1.043	0.56–1.93	0.893
Q5	0.495	0.22–1.11	0.086	3.759	1.06–13.3	0.040
Parental employment (ref. = neither parent employed)						
At least one parent employed	1.290	0.94–1.77	0.113	1.110	0.86–1.44	0.427
Highest parental education (ref. = low)						
Medium	1.330	0.95–1.86	0.095	1.378	1.05–1.80	0.019
High	4.133	2.31–7.41	<0.001	2.635	1.90–3.65	<0.001
Unknown	1.654	1.10–2.48	0.015			
Parental union (ref. = both parents)						
Single parent	0.709	0.52–0.97	0.029	0.767	0.59–0.99	0.043
Housing tenure (ref. = rental)						
Home-owner	0.822	0.32–2.14	0.687	1.302	0.92–1.85	0.140
*N*	2,248			3,897		

Data sources: System of Social Statistical Datasets, Statistics Netherlands and Migrant Trajectories, Statistics Sweden (own calculations).

**Table B2.** Odds ratios (OR) propensity-weighted logistic regression completion of secondary education age 21 for children born in deprived neighbourhoods in Amsterdam and Stockholm metropolitan area including interaction-effects for mobility and family stability (Model 3).

Variable	OR	95% CI	*p*
	Amsterdam		
Type of mobility (ref. = stayers)			
Low mobility short exposure	1.262	0.78–2.03	0.338
Low mobility long exposure	1.195	0.80–1.78	0.381
High mobility	0.926	0.44–1.97	0.841
Gender (ref. = male)			
Female	1.899	1.48–2.43	<0.001
Age parental separation (ref. = no parental separation)			
Age 0–5	0.740	0.42–1.31	0.305
Age 6–12	1.019	0.56–1.84	0.951
Age 13–16	1.232	0.54–2.82	0.621
Family stability throughout childhood (ref. = intact family)			
Non-intact family	1.383	0.69–2.78	0.362
Type of mobility (ref. = stayers) × family stability (ref. = intact family)			
Low mobility short exposure × non-intact family	0.649	0.32–1.33	0.236
Low mobility long exposure × non-intact family	0.687	0.37–1.28	0.239
High mobility × non-intact family	0.615	0.25–1.54	0.298
Parental migrant background (ref. = no migrant parent)			
At least one migrant parent	1.060	0.79–1.42	0.697
Standardised household income quintiles (ref. = Q1)			
Q2	1.168	0.87–1.57	0.307
Q3	1.203	0.82–1.77	0.348
Q4	0.919	0.55–1.53	0.746
Q5	0.863	0.39–1.93	0.720
Parental employment (ref. = neither parent employed)			
At least one parent employed	1.372	1.04–1.81	0.025
Highest parental education (ref. = low)			
Medium	1.299	0.97–1.74	0.077
High	3.274	1.99–5.38	<0.001
Unknown	1.431	1.03–1.99	0.034
Parental union (ref. = both parents)			
Single parent	0.758	0.47–1.22	0.254
Housing tenure (ref. = rental)			
Home-owner	0.775	0.29–2.07	0.610
*N*	2,248		
	Stockholm		
Type of mobility (ref. = stayers)			
Low mobility short exposure	2.391	1.41–4.05	0.001
Low mobility long exposure	1.150	0.78–1.70	0.482
High mobility short exposure	0.784	0.43–1.42	0.422
High mobility long exposure	1.569	0.79–3.10	0.195
Gender (ref. = male)			
Female	1.349	1.10–1.66	0.005
Age parental separation (ref. = no parental separation)			
Age 0–5	1.249	0.76–2.04	0.376
Age 6–12	1.372	0.84–2.25	0.208
Age 13–16	1.980	1.05–3.75	0.036
Family stability throughout childhood (ref. = intact family)			
Non-intact family	0.690	0.36–1.31	0.256
Type of mobility (ref. = stayers) × family stability (ref. = intact family)			
Low mobility short exposure × non-intact family	0.387	0.19–0.81	0.012
Low mobility long exposure × non-intact family	0.626	0.36–1.08	0.089
High mobility short exposure × non-intact family	1.448	0.68–3.08	0.336
High mobility long exposure × non-intact family	0.356	0.16–0.78	0.010
Parental migrant background (ref. = no migrant parent)			
At least one migrant parent	1.518	1.20–1.92	<0.001
Standardised household income quintiles (ref. = Q1)			
Q2	1.177	0.89–1.55	0.245
Q3	1.367	0.92–2.03	0.124
Q4	0.982	0.51–1.91	0.958
Q5	3.236	0.90–11.6	0.071
Parental employment (ref. = neither parent employed)			
At least one parent employed	1.105	0.85–1.43	0.448
Highest parental education (ref. = low)			
Medium	1.387	1.06–1.82	0.019
High	2.616	1.87–3.65	<0.001
Parental union (ref. = both parents)			
Single parent	1.109	0.73–1.69	0.631
Housing tenure (ref. = rental)			
Home-owner	1.290	0.90–1.84	0.162
*N*	3,897		

*Data sources:* System of Social Statistical Datasets, Statistics Netherlands and Migrant Trajectories, Statistics Sweden (own calculations).

## Appendix C. Robustness checks

**Table C1.** Odds ratios (OR) unweighted baseline logistic regression (Model 1) and propensity-weighted logistic regression (Model 2) completion of secondary education age 21 for children born in deprived neighbourhoods in Amsterdam and Stockholm metropolitan area. Definition exposure to neighbourhood deprivation: short exposure <6 years; long exposure: ≥6 years.

Variable	Amsterdam			Stockholm		
	OR	95% CI	*p*	OR	95% CI	*p*
	Model 1: Unweighted baseline model					
Type of mobility (ref. = stayers)						
Low mobility short exposure	1.661	1.19–2.33	0.003	2.133	1.51–3.02	<0.001
Low mobility long exposure	1.107	0.85–1.44	0.451	1.020	0.81–1.29	0.871
High mobility short exposure	1.030	0.61–1.74	0.913	1.121	0.79–1.58	0.518
High mobility long exposure	0.602	0.42–0.87	0.008	0.650	0.50–0.84	0.001
Gender (ref. = male)						
Female	2.050	1.68–2.50	<0.001	1.358	1.15–1.60	<0.001
	Model 2: Propensity-weighted model					
Type of mobility (ref. = stayers)						
Low mobility short exposure	1.088	0.71–1.67	0.701	1.799	1.13–2.85	0.013
Low mobility long exposure	0.999	0.74–1.35	0.994	0.911	0.70–1.18	0.479
High mobility short exposure	0.669	0.36–1.23	0.197	0.988	0.62–1.59	0.961
High mobility long exposure	0.582	0.38–0.88	0.011	0.721	0.54–0.96	0.023
Sex (ref. = male)						
Female	1.991	1.45–2.73	<0.001	1.290	1.01–1.64	0.039
Parental migrant background (ref. = no migrant parent)						
At least one migrant parent	0.930	0.66–1.30	0.674	1.602	1.24–2.07	<0.001
Standardised household income quintiles (ref. = Q1)						
Q2	0.902	0.61–1.33	0.598	1.112	0.84–1.48	0.464
Q3	0.945	0.59–1.51	0.815	1.521	1.01–2.30	0.047
Q4	0.843	0.46–1.55	0.582	1.158	0.64–2.11	0.631
Q5	0.620	0.27–1.41	0.255	3.615	1.00–13.0	0.049
Parental employment (ref. = neither parent employed)						
At least one parent employed	1.452	1.02–2.06	0.037	1.273	0.91–1.78	0.159
Highest parental education (ref. = low)						
Medium	1.540	1.07–2.22	0.021	1.200	0.86–1.67	0.279
High	3.674	1.96–6.89	<0.001	2.321	1.57–3.43	<0.001
Unknown	2.024	1.32–3.11	0.001			
Parental union (ref. = both parents)						
Single parent	0.771	0.55–1.08	0.134	0.817	0.60–1.10	0.189
Housing tenure (ref. = rental)						
Home-owner	1.250	0.47–3.31	0.654	1.237	0.83–1.84	0.297
*N*	2,253			3,909		

Data sources: System of Social Statistical Datasets, Statistics Netherlands and Migrant Trajectories, Statistics Sweden (own calculations).

## Notes

1. For the Netherlands, data on employment and social benefits is available from 1999 onwards and data on income from 2003 onwards. For the preceding years, I use data on the first available year to calculate the neighbourhood SES indicators.

2. The few children who do not move and experience short-term exposure to neighbourhood deprivation are excluded.

3. In the Netherlands, 20% of parents has an unknown educational level, and I therefore included a category ‘unknown parental education’. I conducted all analyses excluding parental education, which gave similar results (available upon request).

4. In Sweden owner-occupied housing includes tenant-ownership, in which residents buy a share into cooperative housing.

5. To test the relative importance of moving frequency and exposure to neighbourhood deprivation, an additional unweighted model was conducted with an interaction between residential mobility and exposure to neighbourhood deprivation. Results are in line with the models with a combined measure of residential mobility and exposure to neighbourhood deprivation (see predicted probabilities in Appendix Figure C1).

## References

[bibr1-00420980261442414] Anderson S Leventhal T Newman S , et al. (2014) Residential mobility among children: A framework for child and family policy. Cityscape 16(1): 5–36.

[bibr2-00420980261442414] Andersson E Janssen H Van Ham M , et al. (2023) Contextual poverty and obtained educational level and income in Sweden and the Netherlands: A multi-scale and longitudinal study. Urban Studies 60(5): 885–903.

[bibr3-00420980261442414] Chetty R Hendren N (2018) The impacts of neighborhoods on intergenerational mobility I: Childhood exposure effects. The Quarterly Journal of Economics 133(3): 1107–1162.

[bibr4-00420980261442414] Chetty R Hendren N Katz LF (2016) The effects of exposure to better neighborhoods on children: New evidence from the Moving to Opportunity experiment. The American Economic Review 106(4): 855–902. 10.1257/aer.20150572 29546974

[bibr5-00420980261442414] Clark WA (2016) Life events and moves under duress: Disruption in the life course and mobility outcomes. Longitudinal and Life Course Studies 7(3): 218–239.

[bibr6-00420980261442414] Clark WA Van Ham M Coulter R (2014) Spatial mobility and social outcomes. Journal of Housing and the Built Environment 29(4): 699–727.

[bibr7-00420980261442414] Coley RL Kull M (2016) Cumulative, timing-specific, and interactive models of residential mobility and children’s cognitive and psychosocial skills. Child Development 87(4): 1204–1220. 10.1111/cdev.12535 27223111

[bibr8-00420980261442414] Cordes SA Schwartz AE Stiefel L , et al. (2016) Is neighbourhood destiny? Exploring the link between neighbourhood mobility and student outcomes. Urban Studies 53(2): 400–417.

[bibr9-00420980261442414] Coulton C Theodos B Turner MA (2012) Residential mobility and neighborhood change: Real neighborhoods under the microscope. Cityscape 14(3): 55–89.

[bibr10-00420980261442414] Elder GH Jr Shanahan MJ (2007) The life course and human development. In: Damon W Lerner RM (eds) Handbook of Child Psychology. Wiley, pp. 665–715.

[bibr11-00420980261442414] Eurostat (2022) Statistical Regions in the European Union and Partner Countries: NUTS and Statistical Regions 2021. Publications Office of the European Union.

[bibr12-00420980261442414] Gambaro L Joshi H Lupton R (2017) Moving to a better place? Residential mobility among families with young children in the Millennium Cohort Study. Population, Space and Place 23(8): e2072.

[bibr13-00420980261442414] Garboden PME Leventhal T Newman S (2017) Estimating the effects of residential mobility: A methodological note. Journal of Social Service Research 43(2): 246–261.

[bibr14-00420980261442414] Gennetian LA Sanbonmatsu L Katz LF , et al. (2012) The long-term effects of moving to opportunity on youth outcomes. Cityscape 14(2): 137–167.

[bibr15-00420980261442414] Goldsmith PR Britton ML Reese B , et al. (2017) Will moving to a better neighborhood help? Teenage residential mobility, change of context, and young-adult educational attainment. Urban Affairs Review 53(2): 305–337.

[bibr16-00420980261442414] Griga D Hadjar A (2014) Migrant background and higher education participation in Europe: The effect of the educational systems. European Sociological Review 30(3): 275–286.

[bibr17-00420980261442414] Haandrikman K Costa R Malmberg B , et al. (2023) Socio-economic segregation in European cities. A comparative study of Brussels, Copenhagen, Amsterdam, Oslo and Stockholm. Urban Geography 44(1): 1–36.

[bibr18-00420980261442414] Hango DW (2006) The long-term effect of childhood residential mobility on educational attainment. The Sociological Quarterly 47(4): 631–664.

[bibr19-00420980261442414] Hansson Å Gustafsson J-E (2020) School mobility and achievement for children placed and not placed in out-of-home care. Scandinavian Journal of Educational Research 64(2): 167–180.

[bibr20-00420980261442414] Haugan T Myhr A (2019) Residential mobility, family structure, and completion of upper secondary education–A registry-based cohort study of the Norwegian Adolescent Population. Frontiers in Psychology 10: 2311. 10.3389/fpsyg.2019.02311 PMC6803472 31681105

[bibr21-00420980261442414] Hedman L Van Ham M (2012) Understanding neighbourhood effects: Selection bias and residential mobility. In: Van Ham M Manley D Bailey N , et al. (eds) Neighbourhood Effects Research: New Perspectives. Springer Netherlands, pp. 79–99.

[bibr22-00420980261442414] Hutchings HA Evans A Barnes P , et al. (2013) Do children who move home and school frequently have poorer educational outcomes in their early years at school? An anonymised cohort study. PLoS One 8(8): e70601. 10.1371/journal.pone.0070601 PMC3734306 23940601

[bibr23-00420980261442414] Kemppainen T Elovainio M Kortteinen M , et al. (2020) Involuntary staying and self-rated health: A multilevel study on housing, health and neighbourhood effects. Urban Studies 57(5): 1049–1067.

[bibr24-00420980261442414] Kuyvenhoven J Haandrikman K Costa R (2025) Cumulative disadvantage during childhood: The association between moving and accumulated neighborhood deprivation over a child’s life course in the Netherlands. Journal of Urban Affairs 47(6): 1977–1997.

[bibr25-00420980261442414] Lennon MC Clark WA Joshi H (2016) Residential mobility and wellbeing: Exploring children’s living situations and their implications for housing policy. Longitudinal and Lifecourse Studies 7(3): 197–200.

[bibr26-00420980261442414] Long L (1992) International perspectives on the residential mobility of America’s children. Journal of Marriage and Family 54(4): 861–869.

[bibr27-00420980261442414] McCaffrey DF Griffin BA Almirall D , et al. (2013) A tutorial on propensity score estimation for multiple treatments using generalized boosted models. Statistics in Medicine 32(19): 3388–3414. 10.1002/sim.5753 PMC3710547 23508673

[bibr28-00420980261442414] Metzger MW Fowler PJ Anderson CL , et al. (2015) Residential mobility during adolescence: Do even “upward” moves predict dropout risk? Social Science Research 53: 218–230. 10.1016/j.ssresearch.2015.05.004 PMC4508758 26188449

[bibr29-00420980261442414] Mollborn S Lawrence E Root ED (2018) Residential mobility across early childhood and children’s kindergarten readiness. Demography 55(2): 485–510. 10.1007/s13524-018-0652-0 PMC5898794 29492798

[bibr30-00420980261442414] Morris T (2017) Examining the influence of major life events as drivers of residential mobility and neighbourhood transitions. Demographic Research 36(35): 1015–1038.

[bibr31-00420980261442414] Mulder CH Hooimeijer P (1999) Residential relocations in the life course. In: Van Wissen LJG Dykstra PA (eds) Population Issues: An Interdisciplinary Focus. Springer Netherlands, pp. 159–186.

[bibr32-00420980261442414] Murphey D Bandy T Moore KA (2012) Frequent Residential Mobility And Young Children’s Well-Being. Research Brief 2012-02. Child Trends.

[bibr33-00420980261442414] Nieuwenhuis J Hooimeijer P (2016) The association between neighbourhoods and educational achievement, a systematic review and meta-analysis. Journal of Housing and the Built Environment 31(2): 321–347. 10.1007/s10901-015-9460-7 PMC5748572 29355196

[bibr34-00420980261442414] Nieuwenhuis J Kleinepier T Van Ham M (2021) The role of exposure to neighborhood and school poverty in understanding educational attainment. Journal of Youth and Adolescence 50(5): 872–892. 10.1007/s10964-021-01427-x PMC8043918 33829400

[bibr35-00420980261442414] Sampson RJ Sharkey P (2008) Neighborhood selection and the social reproduction of concentrated racial inequality. Demography 45(1): 1–29. 10.1353/dem.2008.0012 PMC2831380 18390289

[bibr36-00420980261442414] Sampson RJ Sharkey P Raudenbush SW (2008) Durable effects of concentrated disadvantage on verbal ability among African-American children. Proceedings of the National Academy of Sciences 105(3): 845–852. 10.1073/pnas.0710189104 PMC2242679 18093915

[bibr37-00420980261442414] Scanlon E Devine K (2001) Residential mobility and youth well-being: Research, policy, and practice issues. The Journal of Sociology & Social Welfare 28(1): 119–138.

[bibr38-00420980261442414] Sharkey P (2013) Stuck in Place: Urban Neighborhoods and the End of Progress Toward Racial Equality. The University of Chicago Press.

[bibr39-00420980261442414] South SJ Crowder KD Trent K (1998) Children’s residential mobility and neighborhood environment following parental divorce and remarriage. Social Forces 77(2): 667–693.

[bibr40-00420980261442414] Sweet A Harris R Manley D (2018) Better to stay or go? A longitudinal study of mobility over the compulsory educational life course. Applied Spatial Analysis and Policy 12(3): 697–717.

[bibr41-00420980261442414] Tammaru T Van Ham M Marcińczak S , et al. (2015) Socio-Economic segregation in European Capital Cities: East Meets West. Routledge.

[bibr42-00420980261442414] Tarkiainen L Kemppainen T Lahtinen H , et al. (2024) The effect of cumulative childhood exposure to neighbourhood socio-economic disadvantage on school performance—A register-based study on neighbourhoods, schools, and siblings. European Sociological Review 40(3): 403–416.

[bibr43-00420980261442414] Tønnessen M Telle K Syse A (2016) Childhood residential mobility and long-term outcomes. Acta Sociologica 59(2): 113–129.

[bibr44-00420980261442414] Tucker CJ Marx J Long L (1998) “Moving on”: Residential mobility and children’s school lives. Sociology of Education 71(2): 111–129.

[bibr45-00420980261442414] Vidal S Baxter J (2018) Residential relocations and academic performance of Australian children: A longitudinal analysis. Longitudinal and Life Course Studies 9(2): 133–156.

[bibr46-00420980261442414] Vogel M Zwiers M (2018) The consequences of spatial inequality for adolescent residential mobility. Social Sciences 7(9): 164.

[bibr47-00420980261442414] Wodtke GT Harding DJ Elwert F (2011) Neighborhood effects in temporal perspective: The impact of long-term exposure to concentrated disadvantage on high school graduation. American Sociological Review 76(5): 713–736. 10.1177/0003122411420816 PMC3413291 22879678

